# The ‘inflammatory’ control of hematopoietic stem cells

**DOI:** 10.18632/oncotarget.4591

**Published:** 2015-06-24

**Authors:** Chozha V. Rathinam

**Affiliations:** Columbia University Medical Center, New York, NY, USA

**Keywords:** Immunology and Microbiology Section, Immune response, Immunity

Hematopoiesis is a very complex and well orchestrated process through which all the cells of the immune system are generated. It requires immediate ‘turning on’ and ‘turning off’ of various cytokine receptors, signal transducers, transcription factors and cell cycle inhibitors. While transcriptional regulation of hematopoiesis has been studied, relatively, to a greater extent, role of post-translation modification (PTM) of proteins in the regulation of hematopoiesis remains largely unknown. Although more than 200 forms of protein modifications are identified in mammalian cells, the biological significance of these PTMs has not been understood. Among the various types of protein modifications, ubiquitylation has gained much attention, especially in the field of immunology and stem cell biology, due to its critical roles in the maintenance of a healthy and balanced immune system.

Ubiquitylation (addition of ubiquitin molecules) of proteins is executed by the combinatorial actions of various ubiquitin ligases (enzymes that add ubiquitin) and deubiquitinases (enzymes that remove ubiquitin). Our previous studies identified a key role for E3 ubiquitin ligases, c-Cbl and Itch, in the development and functions of normal hematopoietic stem cells (HSCs) [1–3], and in the restriction of Leukemic Stem Cells [4]. These lines of investigation now open a new avenue for understanding the regulation of intracellular signaling networks that control the development of the immune system from HSCs [5]. To further gain insights into this important mode of HSC regulation, we recently unraveled the role of a deubiquitinase-A20 in HSCs [6].

A20 (also known as Tnfaip3) is a potent anti-inflammatory signaling molecule that restricts multiple intracellular signaling cascades [7]. A20 is a 90 kDa protein that belongs to the ovarian tumor (OTU) family of deubiquitylases (DUB). A noteworthy feature of A20 is that it contains an amino (N)-terminal cysteine protease/DUB domain, that is necessary for the deubiquitylating functions, and a carboxyl (C)-terminal zinc finger (ZNF) domain, that is critical for its E3 ubiquitin ligase functions. Based on this unique capacity, A20 has been classified as a dual function ubiquitin editing enzyme. A20 has been shown to play a critical role in K48-linked ubiquitylation of target proteins, an action that directs target proteins for proteasomal degradation. In addition, A20 removes K63-linked ubiquitin chains from its target proteins (through its DUB activity), which results in cessation of the signaling cascades mediated by the target proteins and in progression of K48-linked ubiquitylation and degradation.

**Figure 1:**
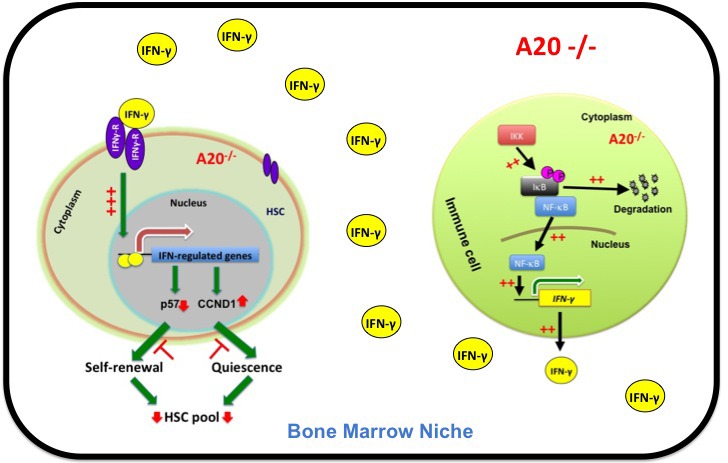
Graphical depiction of the hematopoietic phenotype of A20 deficient mice A20 deficiency results in deregulated Ifn-γ expression in the effector cells of the immune system, due to augmented NF-κB binding to the Ifn-γ promoter. As a functional consequence, Ifn-γ signaling is increased in HSCs, which results in loss of quiescence, functions and premature exhaustion of HSCs.

In an attempt to decipher the role of the ubiquitin editing enzyme-A20 in HSC biology, we generated mice that specifically lack A20 in HSCs and in their progeny (A20_Hem-KO_)[6]. Strikingly, our analysis revealed that a loss of A20 in the hematopoietic system causes reduced body size, cachexia and postnatal lethality [6]. Analysis of the bone marrow (BM) from 14 day old mice indicated decreased BM cellularity, complete loss of LT-HSC pool, increased ST-HSC frequencies and reduced MPP numbers. Strikingly, transfer of total BM from A20_Hem-KO_ mice into lethally irradiated wildtype recipient mice failed to provide radioprotection and engraftment after two weeks of transplantation. Our studies on A20_Hem-KO_ mice indicated increased levels of pro-inflammatory cytokines, such as TNFα, IL1, IL6 and Ifn-γ mRNA in the BM and spleen. Based on the previous findings, we focused on the role of IFNγ in the HSC phenotype of A20 deficient mice. Crossing A20_Hem-KO_ mice with the Ifn-γ reporter (GREAT) mice indicated increased expression of Ifn-γ in both T cells and myeloid cells. Mechanistically, hematopoietic cells of A20_Hem-KO_ mice showed increased binding of NF-κB to its target site in the promoter of the *Ifn-γ* gene. Interestingly, blocking Ifn-γ expression in A20_Hem-KO_ mice resulted in a partial rescue of the HSC phenotype. A summary of the key findings of our recent study on A20 is shown in Figure 1.

While our studies provided strong genetic evidence on the involvement of A20 and pro-inflammatory cytokines in the physiology of HSCs, there are still many interesting questions that remain unanswered and await further investigation. A few of them are discussed below; 1. A large body of work has been done regarding the role of A20 in the immune system, however, most of them were focused on the importance of A20 in the control of NF-κB signals. What are the other protein targets of A20 in the immune cells ?, 2. A20 has been identified as an ubiquitin editing enzyme with both E3 ligase and deubiquitinase functions. Currently, the physiological relevance of these dual functions remains unclear. Does A20 mediated regulation of hematopoiesis require its dual functions ?, 3. Our data provide compelling genetic evidence for an involvement of Ifn-γ signals in the hematopoietic phenotype of A20 deficient mice. However, our studies also suggested the involvement of other signaling pathways in the phenotype, as ablation of Ifn-γ signals resulted only in a partial rescue. What are the roles of other inflammatory cytokines/signals in the hematopoietic phenotype caused by A20 deficiency ?, and 4. We demonstrated a cell extrinsic role of A20 in the control of hematopoiesis. Does A20 control hematopoiesis through cell intrinsic mechanisms ?

Recent studies from several groups have linked germline single nucleotide polymorphisms (SNPs) of *TNFAIP3* with susceptibility to a spectrum of autoimmune disorders in humans [7]. These links to human diseases provide a compelling rationale for further investigation into the mechanisms by which A20 regulates immune cell development and functions.
